# Moderate Autophagy Inhibits Vascular Smooth Muscle Cell Senescence to Stabilize Progressed Atherosclerotic Plaque via the mTORC1/ULK1/ATG13 Signal Pathway

**DOI:** 10.1155/2017/3018190

**Published:** 2017-06-21

**Authors:** Zhenli Luo, Wenhuan Xu, Sai Ma, Hongyu Qiao, Lei Gao, Ran Zhang, Bo Yang, Ya Qiu, Jiangwei Chen, Ming Zhang, Bo Tao, Feng Cao, Yabin Wang

**Affiliations:** ^1^Department of Cardiology, Xijing Hospital, Fourth Military Medical University, Xi'an 710032, China; ^2^Training and Postgraduate Management Department, Medical Administrative Division, Chinese PLA General Hospital, Beijing 100853, China; ^3^Department of Cardiology, Chinese PLA General Hospital, Beijing 100853, China

## Abstract

In order to investigate the effects of autophagy induced by rapamycin in the development of atherosclerosis plaque we established murine atherosclerosis model which was induced in ApoE^−/−^ mice by high fat and cholesterol diet (HFD) for 16 weeks. Rapamycin and 3-Methyladenine (MA) were used as autophagy inducer and inhibitor respectively. The plaque areas in aortic artery were detected with HE and Oil Red O staining. Immunohistochemical staining were applied to investigate content of plaque respectively. In contrast to control and 3-MA groups, rapamycin could inhibit atherosclerosis progression. Rapamycin was able to increase collagen content and a-SMA distribution relatively, as well as decrease necrotic core area. Then we used MOVAS and culture with ox-LDL for 72 h to induce smooth muscle-derived foam cell model in vitro. Rapamycin and 3-MA were cultured together respectively. Flow cytometry assay and SA-*β*-Gal staining experiments were performed to detect survival and senescence of VSMCs. Western blot analysis were utilized to analyze the levels of protein expression. We found that rapamycin could promote ox-LDL-induced VSMCs autophagy survival and alleviate cellular senescence, in comparison to control and 3-MA groups. Western blot analysis showed that rapamycin could upregulate ULK1, ATG13 and downregulate mTORC1 and p53 protein expression.

## 1. Introduction

The rupture of vulnerable plaque followed by thrombus formation is a fatal complication of atherosclerosis. Vulnerable plaque is characterized by a large necrotic lipid core containing infiltrated inflammatory cells and a thin fibrous cap with lower vascular smooth muscle cells (VSMCs) and extracellular matrix. Biological behavior (e.g., senescence, apoptosis, autophagy, and proliferation) of VSMCs is a major contributor to atherosclerotic plaque progression and vulnerability [1]. It is well recognized that senescent VSMCs exhibit impaired proliferation and self-repairing capacity, which reduced fibrous cap formation [2]. In unstable advanced atherosclerotic lesions obtained from patients, senescent VSMCs are found in fibrous caps and exhibit significant loss of the proliferative capability, accompanied by an increase in several senescent markers including enhanced senescence-associated *β*-galactosidase (SA-*β*-gal) activity [3].

Autophagy or cellular self-digestion, the basic catabolic mechanism that involves degradation of unnecessary or dysfunctional cellular components through the actions of lysosomes, has been reported to be implicated in a broad spectrum of mammalian diseases. The mammalian target of rapamycin (mTOR), a serine-threonine kinase as part of the mTORC1 complex, regulates cell's growth, proliferation, autophagy, and metabolism by integrating signals from nutrients, energy status, and growth factors. It is reported that mTOR dysregulation results in cancer, diabetes, and cardiovascular diseases [4]. Furthermore, mTOR is also involved in atherosclerotic plaque progression and destability [5]. A series of clinical trials showed that the mTOR inhibitor rapamycin was used as a drug-eluting stent for atherosclerosis intervention and prevent against stent restenosis by inhibiting VSMC proliferation and migration [6]. However, the effects of mTOR on VSMC autophagy, senescence, and proliferation are complicated. Autophagosome formation requires a series of autophagy-related protein (Atg). The ULK complex composed of ULK1, Atg13, and FIP 200 is one of the most upstream factors of autophagosome in mammals. Therefore, we hypothesized that a moderate dose of rapamycin was capable of inducing autophagy in VSMCs, thus suppressing the progression of atherosclerotic plaques, which is related to regulation of the mTORC1/ULK1/ATG13 signal pathway.

## 2. Methods

### 2.1. Animal Model

Female ApoE^−/−^ mice (8 weeks old) (Jax West Laboratories, West Sacramento, CA, USA) were first fed with a standard laboratory chow diet for one week. Thereafter, all animals were randomly allocated into three groups with *n* = 20 each: (1) control group—mice kept on a standard laboratory chow diet (TD.88137, Harlan Laboratories Inc., Madison, WI) for 16 weeks; (2) high-fat and cholesterol diet (HFD) group—mice were fed with a Western-type diet (containing 15% fat and 0.25% cholesterol) for 16 weeks; (3) low-dose rapamycin group—mice were fed with a Western-type diet and received low-dose rapamycin (50 mg/kg/d) for 16 weeks; (4) high-dose rapamycin group—mice were fed with a Western-type diet and received high-dose rapamycin (100 mg/kg/d) for 16 weeks; and (5) 3-MA group—mice were fed with a Western-type diet and received the autophagy inhibitor 3-MA (100 mg/kg/d) for 16 weeks. All animal procedures were conducted in conformity with the Health Guideline of Chinese PLA General Hospital on the Use of Laboratory Animals, and all experiments were performed in accordance with the Helsinki declaration.

### 2.2. Aortic Tissue Collection

The body weight of mice was measured every week. Blood samples were taken from the inferior vena cava, and animals were sacrificed by euthanasia. Blood was centrifuged to obtain serum. The heart and whole aorta were immediately extracted. The aorta was embedded in optimal cutting temperature (OCT) embedding medium (Tissue-Tek, Sakura Finetek USA, Torrance, CA) for histology and immunofluorescence assay. The remaining aorta was longitudinally opened and fixed with 4% paraformaldehyde for lipid measurement at the surface of the vascular wall. The embedded abdominal aortas were kept at −20°C.

### 2.3. Cell Culture and Experimental Design

The mouse aortic smooth muscle cell line (MOVAS) was purchased from American Type Culture Collection (ATCC) center (Manassas, VA, USA). The cells were cultured in Dulbecco's modified Eagle's medium (DMEM, Gibco, NY, USA), which contained 5 mM glucose and were supplemented with 10% fetal bovine serum (FBS) (Gibco) and 1% penicillin/streptomycin and incubated in 5% CO_2_ at 37°C. The cells were passaged every 2-3 days and then placed into 6-well plates with slides at a density of 4 × 10^5^ cells/cm^2^ and disposed by incubation with ox-LDL (Shanghai Leuven Biological Technology Co.) (80 *μ*g/ml for 72 h). The optimal concentration and time point of ox-LDL were analyzed using Western blot of the autophagy protein marker. The MOVAS cells were randomly divided into the following groups: (1) control group, cells were incubated with DMEM; (2) ox-LDL group, cells were pretreated with ox-LDL (80 *μ*g/ml) for 72 h; (3) ox-LDL + low concentration rapamycin group, ox-LDL-pretreated cells were incubated with the mTOR inhibitor rapamycin (10 ng/ml) for another 24 h; (4) ox-LDL + high-dose rapamycin group, ox-LDL-pretreated cells were incubated with the mTOR inhibitor rapamycin (30 ng/ml) for another 24 h; and (5) ox-LDL + 3-MA group, ox-LDL-pretreated cells were incubated with 3-MA for another 24 hours, respectively. For all data shown, individual experiments were repeated three times.

### 2.4. Protein Extraction and Western Blot Analysis

Total proteins were obtained by rinsing treated cells with ice-cold phosphate-buffered saline (PBS) and lysing in lysis buffer (10 mM Tris, pH 7.4, 20 mM NaCl, 5 mM MgCl2, 0.5% NP-40, and 0.1 mM PMSF). The extracts were then centrifuged at 12,000 rotating speed for 10 min at 4°C, and the clear supernatants containing total protein were collected. The protein concentration was measured with the Bio-Rad protein assay (Blue Skies Biotechnology Company in Shanghai). 20 *μ*l total protein (vessels and cells, resp.) was resolved on SDS-PAGE and transferred onto a nitrocellulose membrane. After blocking in 5% nonfat milk (in Tris-buffered-saline with Tween (TBST)) for 2 hours, membranes were incubated with primary antibodies: mouse anti-rabbit mTOR monoclonal antibody (Cell Signaling, USA), ULK1 monoclonal antibody (Cell Signaling, USA), LC3-II monoclonal antibody (Cell Signaling, USA), Atg5 monoclonal antibody (Merck Millipore Cor., Germany), and *β*-actin monoclonal antibody (Zhong Shan Cor., China), overnight at 4°C. Membranes were washed with TBST for 3 times followed by incubation with the corresponding secondary antibodies. Bands were detected by the use of an enhanced chemiluminescence detection kit (Thermo Electron Corp., Rockford, USA). Expression of individual proteins was normalized to that of *β*-actin. Western blot was repeated at least three times.

### 2.5. Cell Staining for Immunofluorescence Microscopy

Cells were seeded onto slides and incubated with the corresponding intervention, then, respectively, fixed with 4% paraformaldehyde for 15 min at 4°C. After washing with PBS, cells were punched with 1% Triton and then incubated with mouse anti-rabbit LC3-II antibody at 4°C overnight. The slides were washed with PBS and then incubated with mouse anti-rabbit IgG-FITC-conjugated antibody for 2 h at room temperature. Cell nuclei were counterstained by exposure to DAPI (1 mg/ml). After the final wash, the slides were mounted and analyzed under a laser scanning confocal microscope.

### 2.6. Detection of Autophagosomes by TEM Analysis

The cultured MOVAS cells were fixed in 0.1 mol/l sodium cacodylate-buffered (pH 7.4) 2.5% glutaraldehyde solution for 2 h and then rinsed with ice-cold PBS and centrifuged at 1000 ×g for 5 min at room temperature, after which the clear supernatants were removed. Cell pellets were fixed with 2.5% glutaraldehyde in 0.1 M cacodylate buffer (pH 7.4) for at least 30 min at 4°C. After fixation, the specimens were thoroughly washed in 0.1 M cacodylate buffer and then postfixed with 1% osmium tetroxide in the same buffer for 1 h at room temperature. The specimens were dehydrated in a graded series of ethanol and embedded in Epon; then, 0.1 *μ*m thin sections were stained with uranyl-acetate/lead citrate and viewed in TEM (JEM-1230, JEOL, Tokyo, Japan).

### 2.7. Immunohistochemistry

ApoE^−/−^ mice were sacrificed by an intravenous overdose of pentobarbital. The occurrence of plaque rupture and thrombosis of abdominal aorta was observed. Tissue samples (2 cm long) were cut from the abdominal aorta and fixed in 4% formaldehyde. Tissue samples embedded in paraffin were reacted with mouse anti-rabbit CD68 monoclonal antibody (Dako, USA), mouse anti-rabbit *α*-smooth muscle cell (SMC) actin monoclonal antibody (Sigma, USA), Sirius red staining, and Oil red O staining.

### 2.8. SA-*β*-gal Staining

SA-*β*-gal staining was performed as previously described [7]. Briefly, cells were washed twice with PBS followed by fixation with a 3.7% formaldehyde solution for 10 minutes. After washing, cells were then incubated with the SA-*β*-gal staining solution (1 mg/ml X-gal, 40 mmol/l citric acid, 5 mmol/l potassium ferrocyanide, 5 mmol/l potassium ferricyanide, 150 mmol/l sodium chloride, and 2 mmol/l magnesium chloride dissolved in phosphate buffer) (pH 6.0) overnight at 37°C in a CO_2_-free atmosphere. The stained senescent cells were detected by conventional microscopy. Senescent cells were determined by the ratio of SA-*β*-gal-positive cells to the total cell count.

### 2.9. Statistical Analysis

All values are expressed as means ± SEM. Results were analyzed by 1-sample *t*-test versus 100-sample unpaired Student *t*-tests or for multiple comparisons by ANOVA and the Student-Newman-Keuls posttest. Significant differences were accepted when *p* < 0.05.

## 3. Results

### 3.1. Autophagy Activation Inhibits Atherosclerotic Plaque Progression

**Figure 1:**
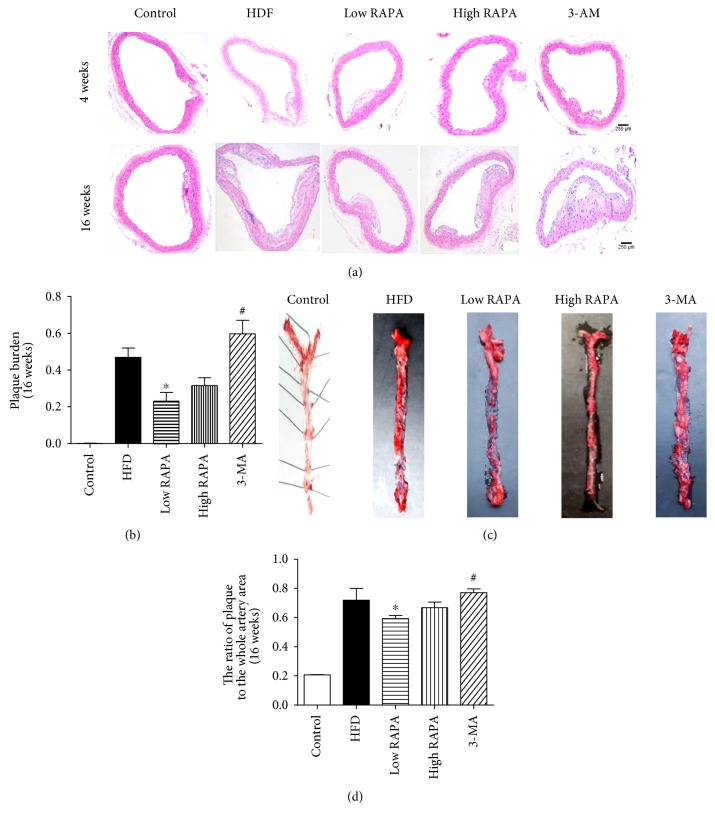
Autophagy activation induced by rapamycin inhibits atherosclerotic plaque progression in ApoE^−/−^ mice. (a) HE staining of an aortic tissue section from ApoE^−/−^ mice fed with HFD for 4 weeks or 16 weeks; (b) quantitation analysis of plaque burden in an aortic tissue section (*n* = 5 per group; ^∗^*p* < 0.05 versus HFD group, ^#^*p* < 0.05 versus low RAPA group); (c) Oil red O staining of the total length of the carotid artery from different mice after 16-week HFD feeding; (d) quantitation of the mean Oil red O-stained plaque area (*n* = 5 per group; ^∗^*p* < 0.05 versus HFD group, ^#^*p* < 0.05 versus low RAPA group).

HE staining of the aortic section revealed that the difference in plaque size was not significant in all groups of ApoE^−/−^ mice fed with an HFD for 4 weeks (Figure 1(a)). However, autophagy agonist rapamycin could significantly decrease the plaque area of ApoE^−/−^ mice fed with an HFD for 16 weeks, in contrast to the HFD group. The autophagy inhibitor 3-MA was able to reverse this trend (Figures 1(a) and 1(b)). Moreover, Oil red O staining of the plaque area to the whole artery demonstrated that rapamycin significantly decreased atherosclerotic lesions of the whole artery, in comparison to the HFD group (*p* < 0.05). These effects were abolished by 3-MA (77.3% ± 0.02% in the 3-MA group versus 59.3% ± 0.01% in the low RAPA group, *p* < 0.05) (Figures 1(c) and 1(d)).

### 3.2. Autophagy Activation Promotes Atherosclerotic Plaque Stability

**Figure 2:**
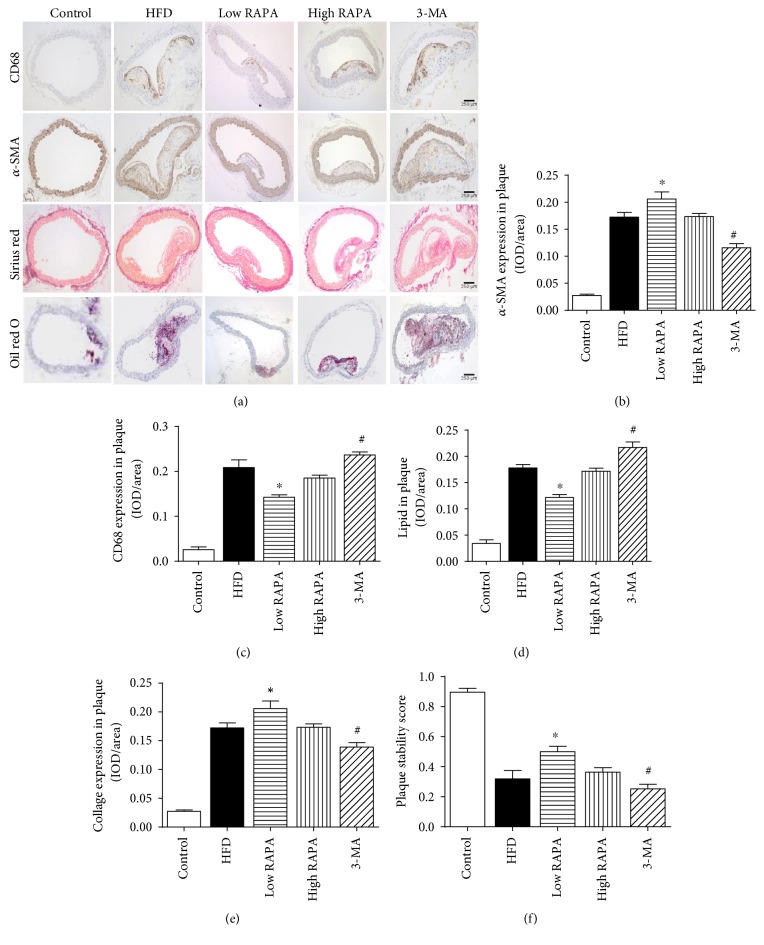
Autophagy activation induced by rapamycin influences the feature of plaque composition in ApoE^−/−^ mice. (a) Immunohistochemistry staining of aortic tissue; (b) the expression of *α*-SMC in plaque by immunohistochemistry staining; (c) the expression of CD68 in plaque by immunohistochemistry staining; (d) the distribution of lipid in plaque by Oil red O staining; (e) the distribution of collagen in plaque by Sirius red staining; (f) quantitative analysis of plaque stability which was calculated as the value of (*α*-SMC area + collagen area)/(macrophage area + lipid area), (*n* = 5 per group; ^∗^*p* < 0.05 versus HFD group, ^#^*p* < 0.05 versus low RAPA group).

A marked change of atherosclerotic plaque morphology was also observed in immunohistochemical staining of carotid tissue isolated from ApoE^−/−^ mice (Figure 2(a)). Further quantification analysis showed that low-dose rapamycin treatment markedly reduced the percent of CD68 expression in the whole plaque, compared with the HFD group, whereas 3-MA abolished the effects of rapamycin (*p* < 0.05) (Figure 2(c)). Similarly, low-dose rapamycin also significantly decreased the necrotic core area. However, the ratio of *α*-SMA and collagen distribution in plaque increased noticeably in the low-dose rapamycin treatment group than in other groups (*p* < 0.05) (Figures 2(b), 2(d), and 2(e)). Further plaque stability analysis by calculating the ratio of collagen content and the necrotic core area revealed that low-dose rapamycin significantly increased plaque stability, compared with HFD and 3-MA groups (*p* < 0.05) (Figure 2(f)).

### 3.3. Autophagy Activation Regulates VSMC Senescence and Cell Cycle

**Figure 3:**
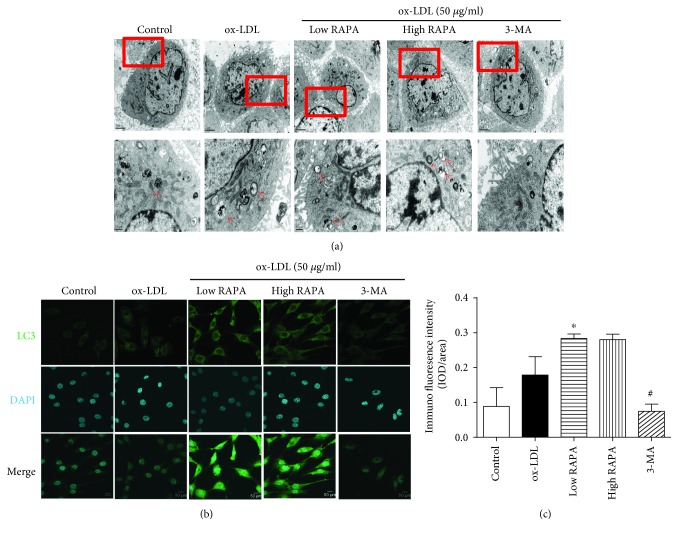
Typical features of autophagy induced by rapamycin in VSMCs. (a) Transmission electron microscopy (TEM) showed autophagosome formation in VSMCs. (b) Immunofluorescence staining revealed LC3II distribution in VSMCs. (c) Quantitative analysis of LC3II expression in VSMCs (*n* = 5 per group; ^∗^*p* < 0.05 versus HFD group, ^#^*p* < 0.05 versus low RAPA group).

Transmission electron microscopy (TEM) and immunohistochemical results revealed that VSMCs treated with low-dose rapamycin exhibited typical features of enhanced autophagy such as increased autophagosome formation and expressions of LC3 II. Nevertheless, 3-MA reversed autophagy in VSMCs induced by rapamycin (Figures 3(a), 3(b), and 3(c)).

**Figure 4:**
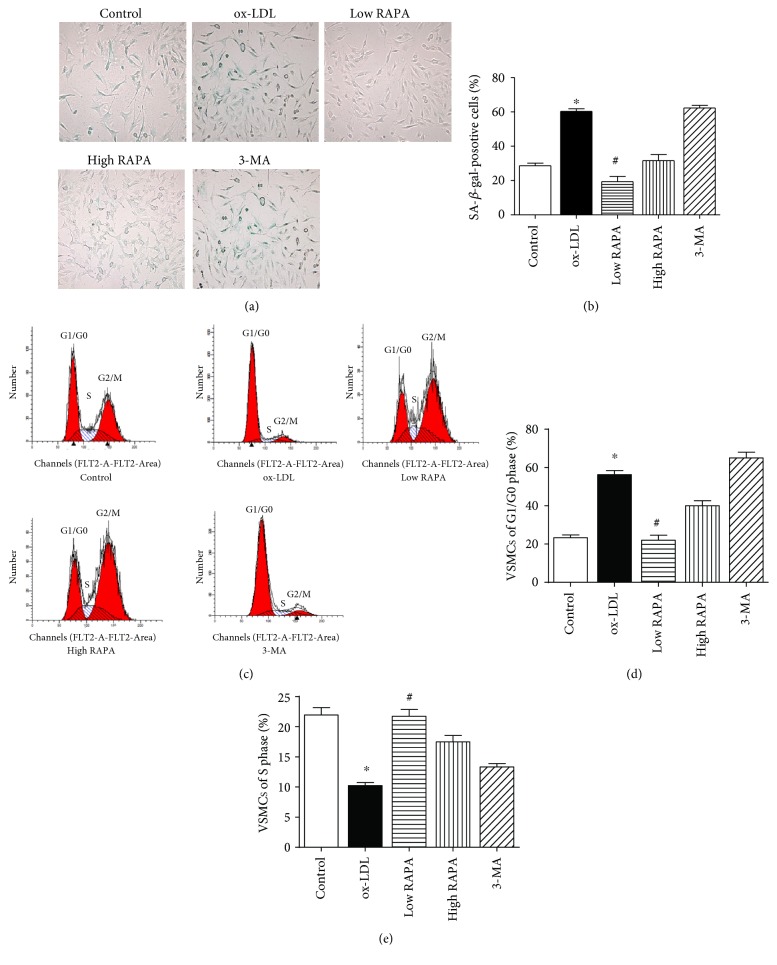
Autophagy activation regulates VSMC senescence and cell cycle. (a, b) Staining of senescence-associated galactosidase (SA-*β*-gal) in VSMCs. (c, d, e) Flow cytometry analysis of cell cycle in VSMCs.

**Figure 5:**
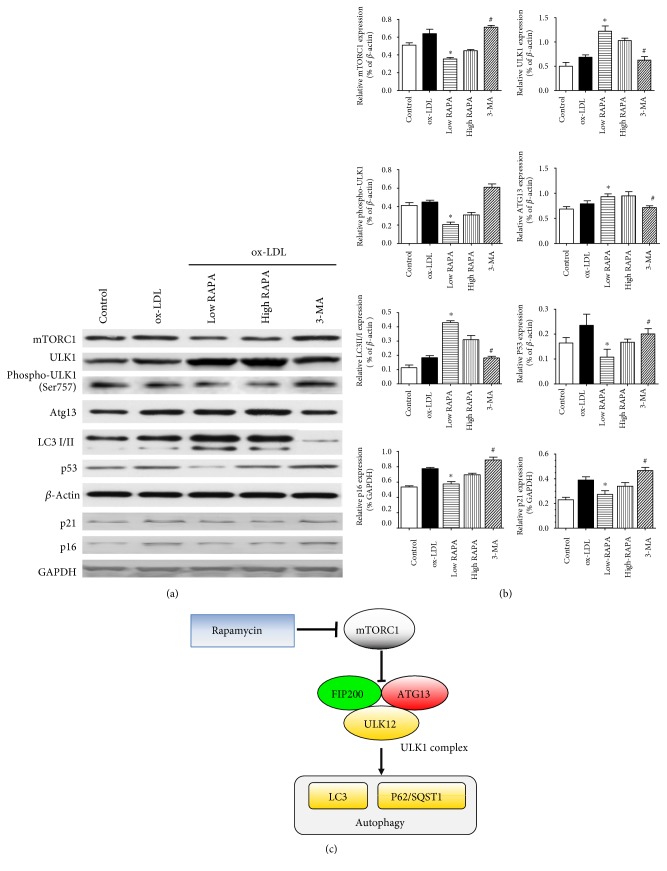
Western blot analysis showed that VSMC autophagy and senescence are regulated via the mTORC1/ULK1/ATG13 signaling pathway.

At 72 hours post-ox-LDL exposure, VSMCs became enlarged and flattened morphologically with strong positive staining of senescence-associated galactosidase (SA-*β*-gal). Low-dose rapamycin treatment could significantly reduce the expression of galactosidase in VSMCs. However, 3-MA reversed the effects of rapamycin (*p* < 0.05) (Figures 4(a) and 4(b)). Western blot analysis revealed that low-dose rapamycin could significantly decrease the expression of senescence markers p16 and p21 in contrast to the ox-LDL group (Figures 5(a) and 5(b)). Flow cytometry data revealed that low-dose rapamycin facilitated VSMC viability by promoting cell cycle progression, compared with ox-LDL and 3-MA groups (*p* < 0.05) (Figures 4(c), 4(d), and 4(e)).

### 3.4. Rapamycin Regulates VSMC Autophagy and Senescence via the mTORC1/ULK1/ATG13 Signaling Pathway

To further investigate the possible mechanisms of VSMC autophagy and senescence, we assessed the level of autophagy-related proteins mTORC1, ULK1, and ATG13. Western blot results revealed that low-dose rapamycin significantly decreased the expression of mTORC1 but increased the protein levels of ULK1 and ATG13 in VSMCs, in contrast to the ox-LDL-induced group (*p* < 0.05). Meanwhile, rapamycin attenuated the phosphorylation level of ULK1. Additionally, low-dose rapamycin was able to inhibit p53 protein expression by inactivating mTORC1. However, 3-MA also abolished the effects of rapamycin on these protein expressions (Figures 5(a) and 5(b)).

## 4. Discussion

VSMCs are a fundamental constitute of atherogenesis. Previous studies showed VSMC proliferation was predominant in atherosclerosis progression, while apoptosis and senescence of VSMCs decreased the fibrous cap and led to plaque instability. Autophagy, which is a major modulator of cellular metabolism, plays crucial roles in the balance of cells' proliferation and apoptosis. However, effects of different levels of autophagy on death, survival, and related mechanism are still not well known. The present study investigates whether moderate autophagy can inhibit VSMC senescence and stabilize atherosclerotic plaque.

We demonstrated moderate autophagy induced by a low dose of rapamycin could inhibit atherosclerosis progression, in contrast to the autophagy inhibitor 3-MA. Moreover, histology results indicated that low-dose rapamycin decreased lipid core size, while it relatively increased collagen and VSMC distribution in advanced plaque. The effects on the fibrous cap and necrotic core were reversed by the autophagy inhibitor 3-MA. Actually, low level of endogenous autophagy can be observed in plaque tissue of the HFD group; however, it may be not sufficient to prevent atherosclerosis development. Exogenous autophagy induction is necessary to alleviate VSMC apoptosis and plaque instability. Recently, Grootaert et al. reported that defective autophagy of VSMCs by genetic deletion of the essential gene Atg7 accelerated postinjury neointima formation and diet-induced atherosclerosis [8]. This result is partly consistent with our data. Some differently defective and inductive autophagies on plaque progression and vulnerability were both investigated in our study. Additionally, we also found that a high dose of rapamycin did not exhibit an extra protective effect on atherosclerosis progression and stability. These infer that moderate autophagy may play more protective roles.

We further investigated the effect of autophagy on VSMCs' survival. Herein, 80 *μ*g/ml ox-LDL-treated VSMCs for 72 h was used as a cell model in vitro. Accumulating evidence showed that the modest dose of ox-LDL (10–40 *μ*g/ml) promoted VSMC proliferation, whereas ox-LDL with higher level (more than 60 *μ*g/ml) for 72 hours was more likely to induce cell apoptosis [9]. We wonder to investigate whether autophagy reversed the ox-LDL role, and the mTOR inhibitor rapamycin was utilized to induce autophagy. Flow cytometry results showed that moderate autophagy displays a protective role in the survival of ox-LDL-induced VSMCs. However, this trend was abolished by 3-MA. It is also showed that the number of VSMCs which stayed in a G1 phase of cell cycle was much less in low-dose rapamycin treatment groups than in other groups. It suggests that moderate autophagy can be considered a beneficial factor for VSMC growth. It is noteworthy that high-dose rapamycin leads to VSMC cycle arrest in the G1 phase. All these results are in line with previous studies that other types of cells (e.g., macrophages, pancreatic *β* cells, and neurons) are also more susceptible to injury-mediated cell death under defective or excessive autophagy conditions [10, 11]. Moreover, previous research revealed that atherosclerosis progression was associated with increased DNA damage and senescence of VSMCs [12]. Therefore, the relationship between autophagy, survival, and aging was further investigated in our study. SA-*β*-gal staining and Western blot analysis of p21 and p16 also demonstrated that ox-LDL treatment significantly promotes VSMC senescence. The autophagy inhibitor further exacerbated ox-LDL-stimulated senescence. Nevertheless, moderate autophagy induced by low-dose rapamycin was able to attenuate VSMC senescence. It is comprehensible that autophagy is a homeostatic cellular recycling mechanism for degrading injured or dysfunctional cellular organelles and proteins in cells and inhibits cell senescence [13, 14].

The relationship between cellular senescence, autophagy, and mTOR has been suggested in other studies [15]. mTOR is a key regular of cell growth and metabolic homeostasis in cells. Previous researches reported that in mammalian cells, rapamycin acts through an allosteric mechanism that acutely disables the nutrient-sensitive mTORC1, but the second complex mTORC2 is unaffected by acute rapamycin treatment [16]. Our mechanism analysis indicated that mTORC1 could promote phosphorylate Ser757 on ULK1. When mTORC1 is inactivated by rapamycin, the repression of ULK1 and ATG13 is relieved, which allows the formation of an active ATG1-ATG13-ATG17 complex and stimulates autophagy [17]. As for the VSMCs, autophagy activation is able to alleviate senescence. Additionally, it is reported that p53 plays an essential role in cellular senescence and quiescence. We demonstrated that mTORC1 inactivation could repress p53 and inhibit VSMC senescence. These findings indicated that the mTORC1/ULK1/ATG13 pathway was mainly involved in cellular senescence and autophagy.

There are some limitations in this study. Firstly, an autophagy inducer or inhibitor was injected into ApoE^−/−^ mice intraperitoneally to regulate autophagy in an animal model. These administration methods cannot differentiate the exact role of rapamycin on various types of cells in lesions such as macrophages, VSMCs, or endothelial cells. In the next research, transgenic mice treated with Cre-LoxP technology to selectively knock out autophagy-related genes (e.g., ATG5 and ATG13) in VSMCs should be applied to investigate the sophisticated role of autophagy on VSMC behaviors in vivo. Secondly, the crosstalk regulation between senescence and autophagy-related genes needs to be investigated in depth.

In summary, this study demonstrated that moderate autophagy induced by low-dose rapamycin could alleviate atherosclerosis progression and promote plaque stability in the ApoE^−/−^ mice model, which was associated with regulating VSMC autophagy and senescence by the mTORC1/ULK1/ATG13 signal pathway.
